# Insulin-Like Growth Factor 1 (IGF-1) in Parkinson's Disease: Potential as Trait-, Progression- and Prediction Marker and Confounding Factors

**DOI:** 10.1371/journal.pone.0150552

**Published:** 2016-03-11

**Authors:** Felix P. Bernhard, Sebastian Heinzel, Gerhard Binder, Karin Weber, Anja Apel, Benjamin Roeben, Christian Deuschle, Mirjam Maechtel, Tanja Heger, Susanne Nussbaum, Thomas Gasser, Walter Maetzler, Daniela Berg

**Affiliations:** 1 Department of Neurodegeneration, Hertie Institute for Clinical Brain Research (HIH), University of Tuebingen, Tuebingen, Germany; 2 German Center for Neurodegenerative Diseases (DZNE), Tuebingen, Germany; 3 Department of Pediatric Endocrinology, University Children`s Hospital Tuebingen, Tuebingen, Germany; University of Catanzaro Magna Graecia, ITALY

## Abstract

**Introduction:**

Biomarkers indicating trait, progression and prediction of pathology and symptoms in Parkinson's disease (PD) often lack specificity or reliability. Investigating biomarker variance between individuals and over time and the effect of confounding factors is essential for the evaluation of biomarkers in PD, such as insulin-like growth factor 1 (IGF-1).

**Materials and Methods:**

IGF-1 serum levels were investigated in up to 8 biannual visits in 37 PD patients and 22 healthy controls (HC) in the longitudinal MODEP study. IGF-1 baseline levels and annual changes in IGF-1 were compared between PD patients and HC while accounting for baseline disease duration (19 early stage: ≤3.5 years; 18 moderate stage: >4 years), age, sex, body mass index (BMI) and common medical factors putatively modulating IGF-1. In addition, associations of baseline IGF-1 with annual changes of motor, cognitive and depressive symptoms and medication dose were investigated.

**Results:**

PD patients in moderate (130±26 ng/mL; p = .004), but not early stages (115±19, p>.1), showed significantly increased baseline IGF-1 levels compared with HC (106±24 ng/mL; p = .017). Age had a significant negative correlation with IGF-1 levels in HC (r = -.47, p = .028) and no correlation in PD patients (r = -.06, p>.1). BMI was negatively correlated in the overall group (r = -.28, p = .034). The annual changes in IGF-1 did not differ significantly between groups and were not correlated with disease duration. Baseline IGF-1 levels were not associated with annual changes of clinical parameters.

**Discussion:**

Elevated IGF-1 in serum might differentiate between patients in moderate PD stages and HC. However, the value of serum IGF-1 as a trait-, progression- and prediction marker in PD is limited as IGF-1 showed large inter- and intraindividual variability and may be modulated by several confounders.

## 1. Introduction

Biomarkers are defined as a characteristic sign that is objectively measured and evaluated as an indicator of normal biological processes, pathogenic processes, or pharmacological responses [1–3]. For example, biofluid markers may serve as surrogate markers of pathology and symptoms, thereby indicating disease traits, its progression and/or prediction of disease characteristics [1,2].

However, for complex and progressive diseases, such as Parkinson's disease (PD), interindividual heterogeneity of patients regarding genetic background, life-style and environmental factors, disease stage, age of onset and severity of (sub) symptoms, comorbidities, and pharmacological or other interventions are clinically relevant [4,5]. In addition to these interindividual differences of PD patients, methodological issues at the stages of biomarker assessment, processing, analysis, interpretation and consideration of confounding factors (e.g., age, sex, body mass index (BMI), diabetes mellitus, thyroid dysfunction, β-adrenergic medication, depression/anti-depressant medication, inflammatory diseases and cancer) [1,6–9] may challenge the identification of reliable and valid biomarkers in PD.

However, indicators of disease trait, progression and prediction are urgently needed for earlier prediction and personalized treatment strategies. Thus, biomarker variance between PD patients and over time, as well as the effect of potential confounders, should be characterized to increase the diagnostic and prognostic value of PD biomarkers.

One promising PD biomarker is insulin-like growth factor 1 (IGF-1) in serum. IGF-1 has been shown to exert neuroprotective and neuroproliferative effects [10,11] and to play an important role for development, plasticity, neuronal survival and differentiation in the nervous system [12,13]. Several studies have shown neuroanatomical and pathophysiological associations of IGF-1 and PD: (1) high densities of IGF-1 receptors in the substantia nigra (SN), (2) IGF-1 level-dependent increase of survival of embryonic dopaminergic and SN neurons [14,15], and (3) protection of neuronal cells in vitro from dopamine-induced toxicity [16,17]. Thus far, studies have shown higher serum IGF-1 levels in PD patients compared with healthy controls (HC)[9,17,18], but also, non-significant differences [18–20] have been reported. The relationship between IGF-1 levels and disease duration or severity is still unclear due to inconsistent findings [9,18,21,22]. Evidence on longitudinal changes in serum IGF-1, showing its potential as PD prediction and progression marker, is very sparse [18,21]. Moreover, additional confounding factors might affect IGF-1 levels. For example, serum IGF-1 levels have been shown to be negatively correlated with age [23–26] and BMI [27] in healthy adults and are modulated by medical conditions, which occur frequently in the elderly, for example, diabetes mellitus [28,29], depression/anti-depressant medication [30,31] or thyroid dysfunction [32].

In the present study, we aimed to further specify the potential of IGF-1 in serum as a trait-, progression-, and prediction biomarker in PD. Thus, we investigated longitudinal serum IGF-1 data of HC and PD patients for differences at baseline, annual changes in IGF-1, and annual changes in clinical measures of PD in relationship to baseline IGF-1. Here, we considered IGF-1 data of PD patients in early and moderate stages. In addition, we analyzed the effect of several confounders on IGF-1 levels, which potentially limit the value of IGF-1 as a specific, valid and reliable PD biomarker.

## 2. Materials and Methods

### 2.1 Participants

The present study investigated the baseline and longitudinal IGF-1 data of 37 PD patients and 22 healthy controls (HC) as a part of the prospective longitudinal MODEP study (Modeling Epidemiological data to study Parkinson disease progression) [33]. In this study, we included PD patients categorized at baseline to be “early stage” (E-PD: ≤3.5 years disease duration; n = 19) and “moderate stage" (M-PD: 4 to 8 years; n = 18) and HC. Assessments with a comprehensive clinical battery and acquisition of biomaterial were performed every 6 months. In the present study, up to 8 visits in biannual assessments (i.e., over up to 3.5 years) were analyzed. Data of one tremor-dominant PD patient was not included as this subtype might represent a distinct entity within PD, which differs from other subtypes in key biomarkers of PD [21,34]. Thus, in the present study, only the akinetic-rigid and equivalent PD subtypes were included. This study was approved by the ethical committee of the Medical Faculty of the University of Tuebingen (Nr 46/2010). All procedures were performed according to the Declaration of Helsinki in its most recent version, and all subjects provided their written informed consent.

### 2.2 IGF-1 measurements

Blood samples were drawn after fasting the previous night, centrifuged immediately after collection and stored at -70°C until further analysis [35]. For the IGF-1 quantification, validated radioimmunoassays with anti-human IGF-1 antibodies (rabbit) and recombinant human IGF-1 were used [9,36,37].

### 2.3 Confounding factors

Age, sex and body mass index (BMI, kg/m^2^), as well as the presence or absence of other medical factors known to affect IGF-1 levels (hereafter termed “medical confounders”), were assessed. The medical confounders included the following diseases and medication, which have been shown to affect IGF-1 levels: diabetes mellitus (reported in medical history or inferred by antidiabetic medication intake) [28,29], beta-adrenergic medication [9], depression (and/or antidepressant medication) [30,31], neuroleptic medication [38], thyroid dysfunction [32], inflammatory diseases [29,39] and cancer [40].

### 2.4 Clinical progression parameters

PD motor symptom severity was assessed using the Unified Parkinson Disease Rating Scale part 3 (UPDRS-III) [41] and Hoehn and Yahr (HY) stage. Only data assessed the OFF medication state were considered. We calculated the levodopa equivalent dose [LED; mg per day] of medication using standard procedures [42]. Moreover, we assessed depressive symptoms using the Beck Depression Inventory (BDI-II) [43], and global cognitive function using the Mini-Mental State Examination (MMSE) [44].

### 2.5 Statistical analysis

Descriptive statistics are provided as the mean ± standard deviation. We performed analyses of covariance (ANCOVAs) with baseline IGF-1 levels as the dependent variable, diagnosis (HC, PD) as between-subject factor and sex, age, BMI and the presence of medical confounders as covariates. The same ANCOVA with the between-subject factor PD stage (HC, E-PD, M-PD) and subsequent post-hoc t-tests between groups were then performed (while accounting for multiple testing using Bonferroni-corrections of three groups, i.e., p < .017 as threshold of significance). For comparison of categorical variables, chi-squared tests, and for continuous variables, t-tests between groups, were applied. Pearson correlations were obtained. For comparison of differential correlations between groups, a Fisher r-to-z transformation and one-tailed z-tests were performed. IGF-1, UPDRS-III, LED, BDI and MMSE annual changes were derived from linear equations where only individuals with at least three values were considered. Differences in the annual changes of clinical parameters were tested (t-tests) between PD patients with high (n = 18) and low (n = 19; median split at 122 ng/mL) baseline IGF-1 levels. Significance level was set to α = 0.05 (two-tailed) while non-significant statistical trends (p < .1) were also reported.

## 3. Results

### 3.1 Descriptive statistics

The age and sex ratio (females / males) did not differ between PD patients (64 ± 7 years, 15 f / 22 m) and HC (64 ± 7 years, 9 f / 13 m) at baseline (p > .1). In addition, BMI (26 ± 4 in PD, 26 ± 3 in HC; BMI > 30: 2 HC, 3 E-PD, 3 M-PD) and the presence of medical confounders did not differ between groups (12 of 37 PD, 8 of 22 HC; p > .1). With regard to depression/intake of antidepressants (0 HC, 4 E-PD, 6 M-PD), pronounced differences between groups were observed but not for other factors, i.e., diabetes mellitus/anti-diabetic medication (1, 1, 1), beta-adrenergic medication (2, 2, 1), thyroid dysfunction (3, 3, 2), inflammatory disease (0, 1 Morbus Crohn, 0), and cancer (0, 1 breast cancer, 0). While E-PD did not differ from M-PD in sex ratio, BMI or presence of medical confounders (p > .1), M-PD (67 ± 5 years) was significantly older than E-PD (62 ± 8 years, p = .030) and showed more severe PD motor symptoms (UPDRS-III, M-PD: 38 ± 15 (19–68); E-PD: 22 ± 9 (5–32), p = .002; HY, M-PD: 2.7 ± 0.7 (2–4); E-PD: 1.7 ± 0.6 (1–3), p < .001).

### 3.2 IGF-1 as a PD trait marker: IGF-1 at baseline

**Fig 1:**
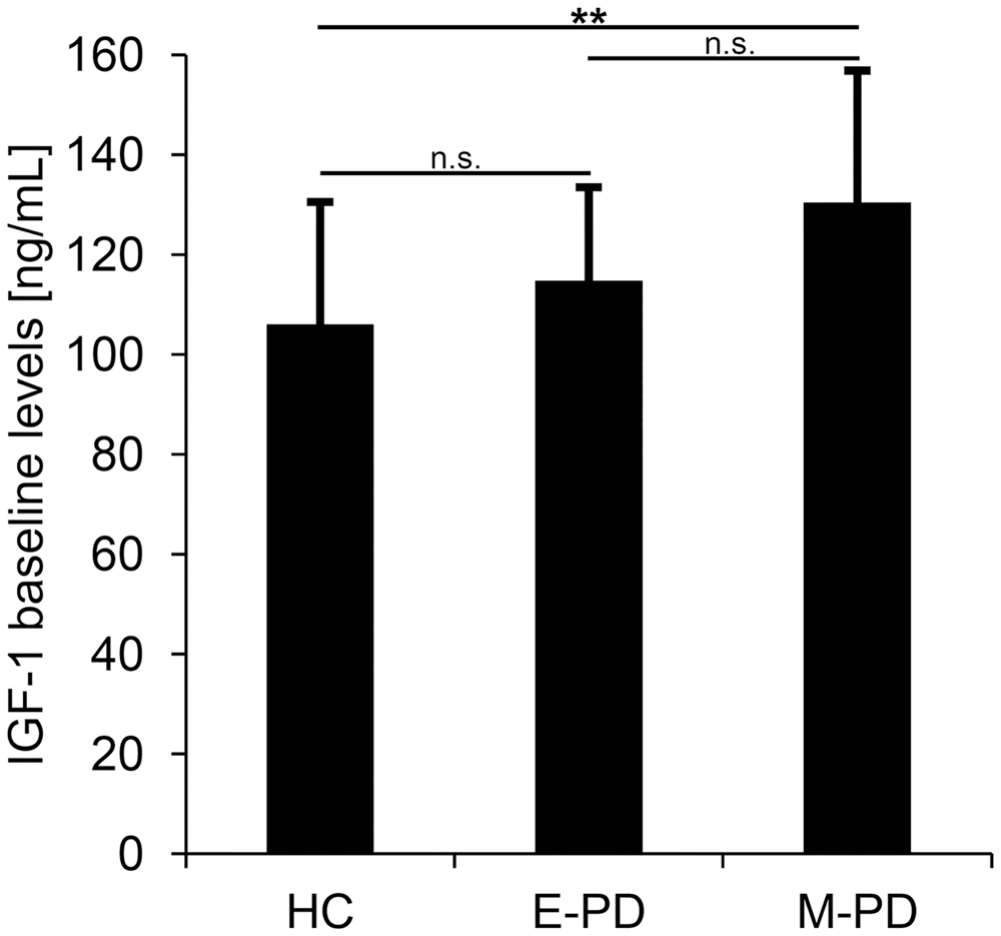
IGF-1 levels at baseline. IGF-1 levels at baseline in healthy controls (HC), early-stage Parkinson's disease (E-PD) and moderate-stage PD (M-PD) adjusted for the effects of covariates age, sex, body mass index and the presence of medical confounders. Error bars indicate standard deviations. Group differences were tested using t-tests and a Bonforroni-corrected threshold of significance (three tests; p = .017). n.s. = non-significant; * p < .017.

Overall, PD patients showed significantly increased IGF-1 baseline levels compared with HC (F_1,53_ = 6.06, p = .017, partial η^2^ = .10; without including covariates p = .026). However, when also considering the PD stage for the grouping of PD patients, only M-PD (130 ± 26 ng/mL, adjusted for covariates, t_38_ = 3.03, p = .004), but not E-PD (115 ± 19 ng/mL, p > .1) showed significantly increased IGF-1 baseline levels compared with HC (106 ± 24 ng/mL). In addition, M-PD showed higher levels than E-PD; however after Bonferroni-correction for multiple testing, this difference was not significant (t_35_ = 2.09, p = .044, see Fig 1).

### 3.3 IGF-1 as a PD progression marker: Annual IGF-1 change

**Fig 2:**
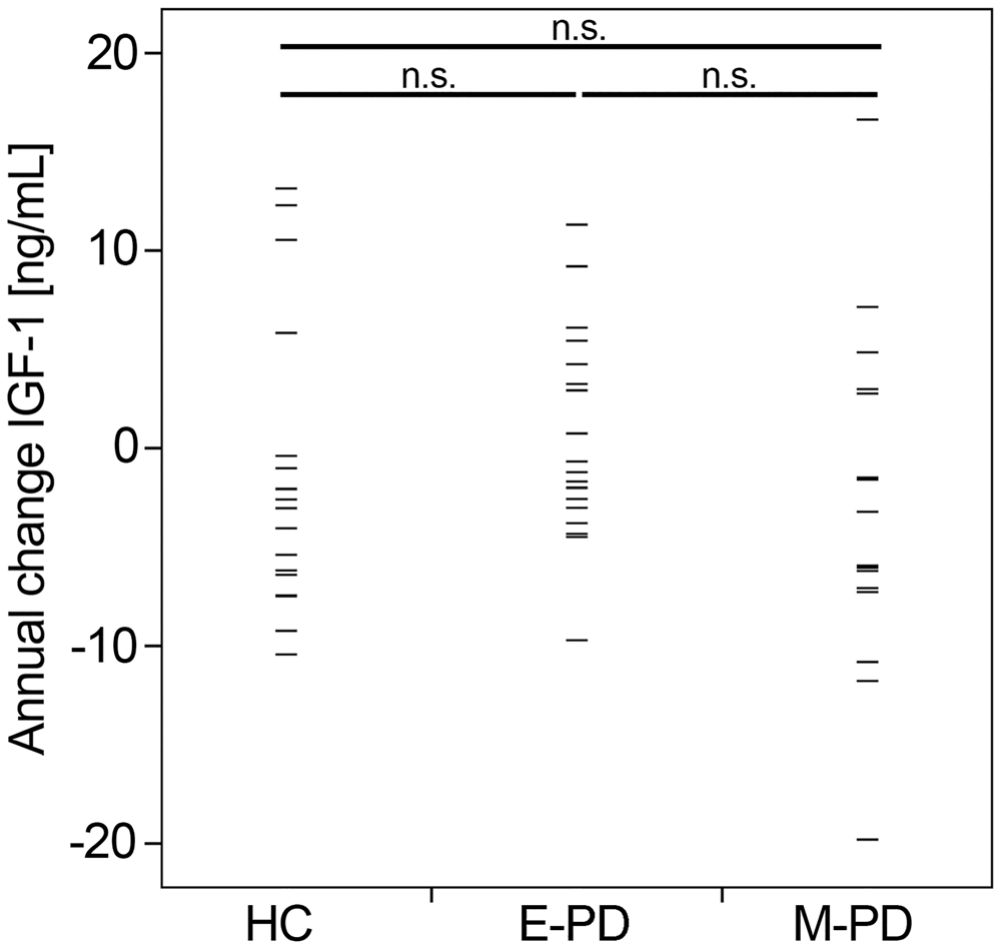
IGF-1 changes over time. Linear annual change in IGF-1 levels of single individuals within groups of healthy controls (HC) and patients with Parkinson's disease in early (E-PD) or moderate (M-PD) disease stages. High inter-individual differences, with increasing (+) and decreasing (-) IGF-1 levels over time, were observed. Differences between groups were not significant (n.s.) as tested using t-tests.

HC (-1.5 ± 7.0 ng/mL annual change in IGF-1 levels; adjusted for effects of covariates at baseline), E-PD (-0.1 ± 6.4), and M-PD (-2.5 ± 8.0) showed no significant differences in IGF-1 changes per year (p > .1). In PD patients, changes in IGF-1 levels were not significantly correlated with the disease duration at baseline (r = -.21, p > .1). Within groups of HC, E-PD and M-PD demonstrated a high standard deviation of annual IGF-1 changes (Fig 2).

### 3.4 IGF-1 as a PD prediction marker: Baseline IGF-1 and annual change of clinical parameters

**Fig 3:**
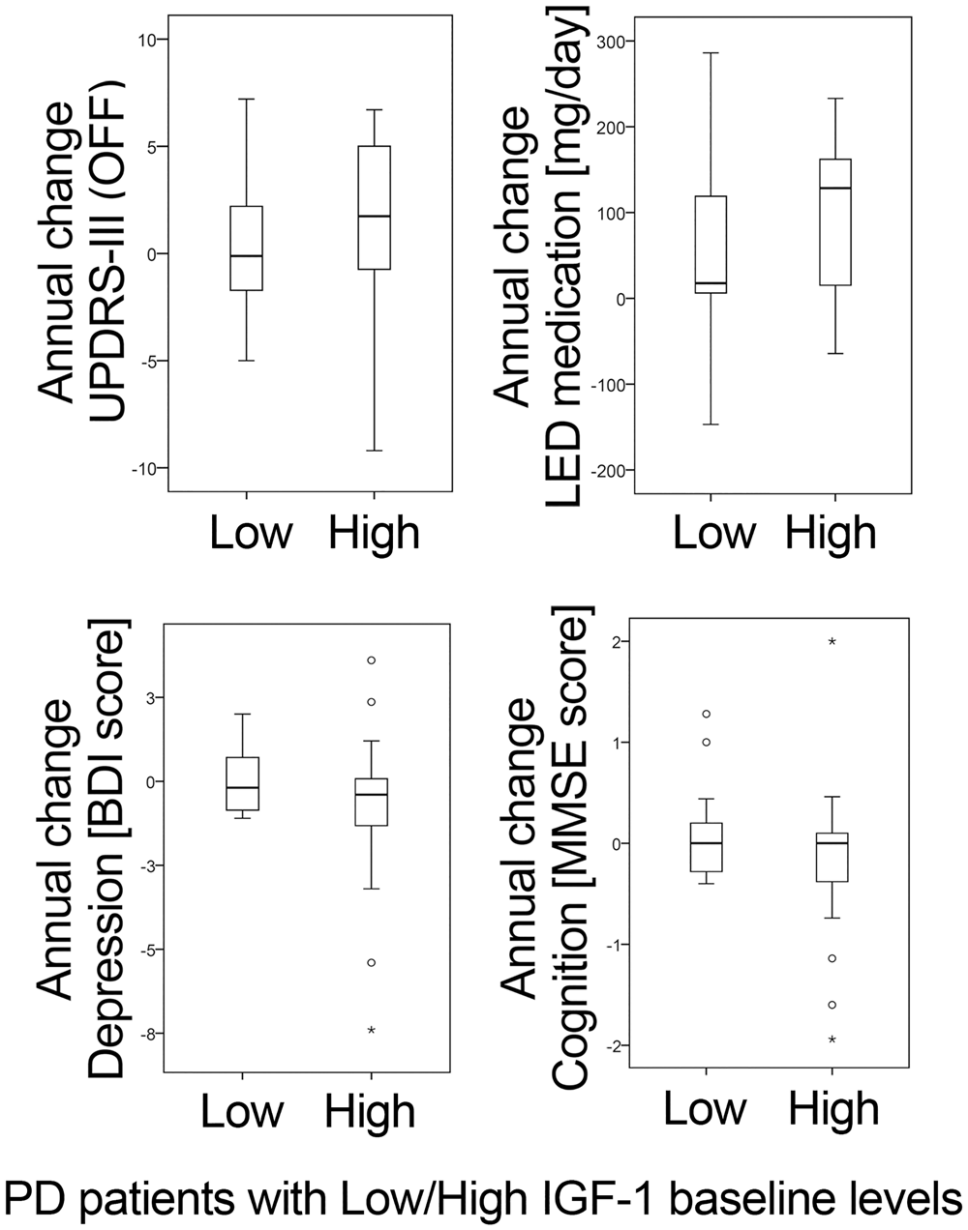
IGF-1 as a predictor of changes in clinical parameters. PD patient groups with low and high IGF-1 baseline levels (median split at 122 ng/mL). Groups show no significant differences (p > .1) in annual changes of (a) PD-motor symptom severity (UPDRS-III), (b) PD-medication (L-dopa equivalent dose, LED), (c) depressive symptoms (BDI score) and (d) global cognitive function (MMSE score).

To investigate the predictive value of IGF-1 regarding longitudinal changes in key clinical parameters of PD, we compared these disease progression characteristics in PD patients with high and low baseline IGF-1 serum levels. PD patient groups with low (<122 ng/mL) and high (≥122 ng/mL) IGF-1 baseline levels differed neither in annual changes of motor symptom severity (UPDRS-III), levodopa equivalent dose medication (LED), depressive symptoms (BDI), nor global cognition (MMSE), p > .1; see Fig 3.

### 3.5 Confounding factors of IGF-1

In the overall cohort, age did not affect IGF-1 baseline levels (r = -.01, p > .1). However, PD patients and HC differed significantly in age-IGF-1 correlation (z = -1.99, p = .023). Here, HC showed a significant negative correlation (r = -.47, p = .028), whereas PD patients showed no correlation (r = -.06, p > .1). Overall, BMI was negatively correlated with baseline IGF-1 levels (r = -.28, p = .034); however, correlations were not significant in the separate diagnostic groups (PD: r = -.30, p = .069; HC: r = -.31, p > .1). Sex had no impact on baseline IGF-1 levels (p > .1). The presence of medical confounders showed no significant effect on IGF-1 levels, neither in the overall nor in separate diagnostic groups (p > .1), individuals with these factors numerically showed lower IGF-1 levels compared with those without these factors (PD with: 116 ± 26, without: 125 ± 27; HC with: 100 ± 12, without: 110 ± 26 ng/mL). These potential medical confounders showed no significant intercorrelations or dependencies in the overall or diagnostic groups (p > .1).

## 4. Discussion

In the present study, we investigated the characteristics of IGF-1 in serum important for a valid and reliable biomarker in Parkinson's disease (PD). Specifically, we evaluated the potential of IGF-1 (1) in differentiating PD patients in early and moderate stages from healthy controls (HC, trait marker), (2) for indicating differences in longitudinal IGF-1 changes in PD and HC (progression marker), and (3) for its prediction of changes in PD symptoms over time (prediction marker). Moreover, we analyzed several putative medical confounders of IGF-1, which occur frequently in elderly individuals and discuss the specificity of IGF-1 as a PD biomarker.

### Potential of IGF-1 as a trait marker

We confirmed IGF-1 as a PD trait marker by replicating several previous findings, which showed increased IGF-1 levels in PD patients compared to healthy controls (HC) [9,15,22]. However, in contrast to previous findings [9,18,26,45], PD patients in early disease stages (E-PD, ≤ 3.5 years disease duration) did not show significantly elevated IGF-1 levels. In E-PD (115 ± 19 ng/mL) IGF-1 values were numerically higher compared with HC (106 ± 24 ng/mL). The difference between E-PD and M-PD (p = .044) was not significant after correcting for multiple testing. The lack of statistical significance might partly be due to the small sample size of the present study. The significantly increased IGF-1 levels in moderate stage PD (M-PD, > 3.5 years disease duration; 130 ± 26 ng/mL) compared with HC contradict previous findings [18]. More precisely, a previous study found decreased IGF-1 levels in more severely affected PD patients (Hoehn and Yahr (HY) stage 3–5) compared with more mildly affected PD patients (HY stage 1–2) [18]. However, our findings suggest that the potential of IGF-1 as a PD trait biomarker is most pronounced in advanced disease stages, whereas the distinction between PD patient and HC might be less reliable in early stages. Because there are no longitudinal studies on IGF-1 in PD patients yet available, we defined the two different stages of Parkinson's disease as “early” and “moderate” PD. These two stages are based on previous definitions for “early” PD stages as ≤3.5 years disease duration [26]. There might be differences in the definition of PD stages based on disease duration [9,26] or severity of clinical symptoms, e.g., dependent on HY stages [18], which may complicate comparisons of our results with previously published data. However, we propose that the large inter- and intraindividual variability within all three groups (E-PD, M-PD, HC) at baseline, as well as over time (see below and Confounding factors), indicates that the potential of IGF-1 as a trait marker is limited.

### Potential of IGF-1 as a progression marker

The longitudinal changes in IGF-1 levels in HC and both groups of PD patients (E-PD, M-PD) over (up to) 3.5 years showed no significant differences or a relationship with disease duration, as shown at baseline levels and the annual changes in IGF-1 in each group (Figs 1 and 2). Moreover, large interindividual differences, i.e., increasing, decreasing or stable IGF-1 levels over time, were observed in all three groups. Similar to the variance of IGF-1 levels at baseline, the variance between individuals regarding their intraindividual changes of IGF-1 over time, suggests that IGF-1 is modulated by several different factors. Consequently, IGF-1 does not appear to have relevant potential as a marker indicating distinct progression characteristics in PD.

### Potential of IGF-1 as a prediction marker

“High” and “low” IGF-1 serum levels of PD patients at baseline showed no significant associations with the subsequent changes in motor (UPDRS-III), cognitive (MMSE), psychiatric/depression (BDI) or medical treatment (LED) over (up to) 3.5 years. Consistent with these findings, we assumed that the potential of IGF-1 as a prediction marker in PD is also limited.

### Confounding factors

As exemplified by IGF-1 levels in HC, published studies have shown large absolute and interindividual differences as indicated by the mean ± standard deviation values [ng/mL] in HC: 79.1 ± 23 [21,45], 113 ± 51 [20], 114 ± 5.9 [18], 127 ± 31 [9], 155 ± 36 (plasma) [22], and, in this study: 106 ± 24. These differences suggest that further confounding factors not related to PD or methodological issues modulating measured IGF-1 serum levels complicate the direct comparison of findings of different samples and laboratories.

Increasing age has repeatedly been shown to be associated with reduced serum IGF-1 levels in elderly individuals and in different phases of the adult life. Interestingly, the well-known negative correlation of IGF-1 with age [46,47] could only be shown for HC but not for PD patients. This finding suggests that, due to hidden interactions (e.g., differential age effects in PD/HC), "correcting" for confounding factors of IGF-1 may be statistically challenging; in particular, as several confounding factors frequently occur in the elderly. Consistent with previous findings in a large population-based cohort [27], we showed that BMI correlates negatively with serum IGF-1 (also below the BMI obesity threshold), suggesting that the common exclusion of individuals with obesity (BMI > 30) [9,26,40] might not be sufficient to account for the effect of this factor on IGF-1 levels [40]. Previously, IGF-1 levels in healthy females compared with males have been shown to be lower [27]. However, we found no significant effect of sex on IGF-1 levels after accounting for other confounders; this may be due to sample size and/or other confounders [47]. The presence of putative medical confounders showed no effect on IGF-1 level, which does not correspond to studies showing decreased IGF-1 serum levels in diabetes [28], thyroid dysfunction [32], β-adrenergic medication [9], depression and/or anti-depressant medication [30,31], inflammatory diseases [29,39] and cancer [40]. It may be difficult to statistically detect independent effects of sex or medical confounders in the presence of other effects (e.g., age, BMI, PD). Thus, we cannot exclude the possibility that sex or medical confounders can still physiologically modulate IGF-1 levels.

Apart from these confounding factors, variance in IGF-1 levels in PD may be due to the heterogeneity of PD patients, who often differ in motor, as well as non-motor, symptom constellations. For example, in PD patients with cognitive decline or an additional diagnosis of dementia, IGF-1 levels might show a distinct alteration as suggested by findings showing that IGF-1 levels change differently in Alzheimer's disease (AD) and PD [22,48]. An increased risk to develop AD [48] has been shown to be associated with *lower* IGF-1 serum levels in HC who subsequently converted to AD. Thus, increased IGF-1 serum levels appear to be protective against subclinical and clinical neurodegeneration in AD [48–50]. Detrimental processes modulating IGF-1 levels in PD and AD might complicate the interpretation of IGF-1 levels. In this regard, IGF-1 serum levels were recently shown to be negatively correlated with cognitive performance (MMSE scores) in PD patients [22]. Thus, associations of PD and elevated IGF-1 might best be shown in PD patients without cognitive impairment or β-amyloid pathology.

In conclusion, IGF-1 might serve as a PD trait marker and our results suggest longer disease duration to be associated with more pronounced differences between PD patients and healthy controls. Within PD patients, our prospective study showed no potential of IGF-1 levels as a progression marker or prediction marker in PD. However, additional confounding factors (e.g., age, BMI, and possibly diabetes, infection, dementia and others) may affect IGF-1 and contribute to the generally large variance of IGF-1 within PD patients as well as healthy controls. Thus, the potential of IGF-1 as a reliable and specific PD biomarker is at least limited. The suggested neuroprotective function of IGF-1 [10,11,15,51] and its relevance for individual PD patients should be further investigated to better address future clinical questions in PD regarding IGF-1.
